# Epidemiology of movement disorders in the adult population of Qatar: Prevalence, types, and demographics

**DOI:** 10.5339/qmj.2026.25

**Published:** 2026-06-04

**Authors:** Ali Msheik, Alaaeldin Ahmed, Fatima Al-Sada, Zeinab Al Mokdad, Ibrahim Abdelhafez, Aisha Alkubaisi

**Affiliations:** 1 Neurological Surgery, Neuroscience Institute, Hamad Medical Corporation, Doha, Qatar; 2 Medical Ethics, Lebanese University Faculty of Medical Sciences, Beirut, Lebanon

**Keywords:** Movement disorders, tremors, Parkinson’s disease, dystonia, neuropathy, retrospective study

## Abstract

**Background:**

Movement disorders encompass a range of neurological conditions affecting speed, fluency, quality, and ease of movement and including Parkinson’s disease, essential tremor, dystonia, and others. Understanding their epidemiology is crucial for effective public health planning and resource allocation. This study aims to provide a comprehensive analysis of the epidemiological trends of movement disorders in Qatar.

**Methods:**

This study is a retrospective descriptive epidemiological cross-sectional analysis based on available clinical data from individuals diagnosed with movement disorders at Hamad General Hospital from January 2014 until March 2025. Individuals of any nationality residing in Qatar, diagnosed with any type of movement disorder, and aged 18 years or older at the time of diagnosis were included. The key variables extracted from the medical records included the age at diagnosis, the gender, nationality, and the type of movement disorder of each patient.

**Results:**

A total of 3017 patients were evaluated, with tremors (54%) and Parkinson’s disease (25.8%) being the most common diagnoses; Qataris made up 39% of the cohort. Tremor patients were predominantly male (62%) and under 60 years old, with Qataris and Indians being the largest groups. Parkinson’s patients were mostly male (67%), diagnosed between the ages of 41 and 80 years. Dystonia and neuropathy were less common; dystonia showed a younger median age (45 years), while neuropathy cases, mostly Qataris, had a median age of 70 years.

**Conclusion:**

The findings underscore the influence of age, gender, and nationality on disease distribution. Despite its retrospective nature, the study lays important groundwork for future research. Prospective studies are needed to deepen understanding and support targeted healthcare strategies.

## 1. INTRODUCTION

Movement disorders encompass a range of neurological conditions affecting speed, fluency, quality, and ease of movement. These include Parkinson’s disease (PD), essential tremor, dystonia, and others. Such disorders can lead to significant disability, reduced quality of life, and increased healthcare costs. Understanding their epidemiology is crucial for effective public health planning and resource allocation.^1^

Globally, the prevalence and distribution of movement disorders vary due to genetic, environmental, and demographic factors.^1,2^ A meta-analysis of 42 population-based studies reported a global prevalence of essential tremor at 1.33% across all ages and 5.79% among individuals aged ≥65 years, with a marked increase of 74% per decade of age.^3^ Recent global reports estimate the age-standardized prevalence of PD and dystonia at 139 and 16.4 per 100,000 people, respectively.^4,5^ In the Middle East and North Africa (MENA) region, the age-standardized prevalence of PD in 2019 ranged from 63.6 to 119.3 per 100,000.^2^ Population-based epidemiological data on movement disorders in Gulf Cooperation Council (GCC) countries remain limited, with most available studies derived from hospital-based cohorts or modelled registry data. Qatar has a unique demographic structure characterized by a large expatriate workforce and a relatively young population profile,^6^ warranting local epidemiological evaluation.

This study aims to provide a comprehensive analysis of the epidemiological trends of movement disorders in Qatar, leveraging available clinical and demographic data at Hamad General Hospital (HGH), the largest tertiary care center in Qatar, and part of Hamad Medical Corporation (HMC), the principal public healthcare provider in the country. Even though HMC includes multiple hospitals and healthcare facilities across Qatar, only patients evaluated at HGH were included in the present analysis. By examining factors such as prevalence, age, gender distribution, and subtypes of movement disorders, this research contributes to a better understanding of these conditions in the region and supports the development of evidence-based healthcare strategies.

## 2. METHODS

### 2.1 Study Design

This study is a retrospective descriptive epidemiological cross-sectional analysis based on available clinical data from individuals diagnosed with movement disorders at HGH. This study applied descriptive statistical methods to summarize prevalence rates and demographic distributions across movement disorder categories. This study was approved by the HMC Medical Research Centre Institutional Review Board (MRC-01-25-875).

### 2.2 Study Population

The study population included individuals diagnosed with movement disorders, including PD, essential tremors, dystonia, and neuropathy, from January 2014 until March 2025 at HGH. The data were collected from the clinics and the electronic medical records. Individuals aged 18 years or older at the time of diagnosis were included. Pediatric patients (<18 years) were excluded, as the movement disorders clinics at HGH primarily serve adult neurology patients. Pediatric movement disorders are typically managed in separate pediatric neurology clinics, which were not included in this data extraction.

### 2.3 Data Collection

The key variables extracted from the medical records included the age at diagnosis, the gender, nationality, and the type of movement disorder of each patient. For patients diagnosed with tremor, the categorization was based on the primary clinical diagnosis entered into the electronic medical record. Subtypes of tremor (essential tremor, Parkinsonian tremor, dystonic tremor, enhanced physiological tremor) were not consistently documented across all cases and, therefore, were collectively analyzed under the general category of “tremor” to ensure data reliability. Dystonia was identified based on the treating neurologist’s primary diagnosis. Only patients with a confirmed clinical diagnosis of PD, as documented in the electronic medical records, were included in the analysis. Cases diagnosed with other forms of Parkinsonism (progressive supranuclear palsy, multiple system atrophy, corticobasal degeneration, or unspecified Parkinsonism) were excluded to maintain diagnostic specificity. Inferential statistical analysis was not performed, as the primary objective was to characterize the epidemiological burden rather than test specific hypotheses or associations.

## 3. RESULTS

### 3.1 Demographics of the whole cohort

**Figure 1.**
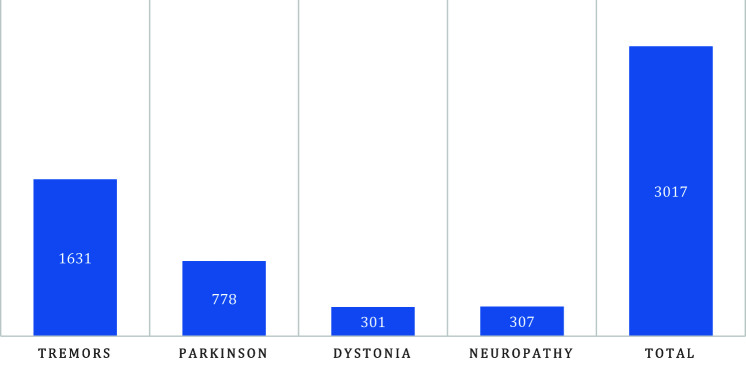
Distribution of adult patients (≥18 years) diagnosed with movement disorders at Hamad General Hospital, Doha, Qatar (January 2014–March 2025).

**Figure 2.**
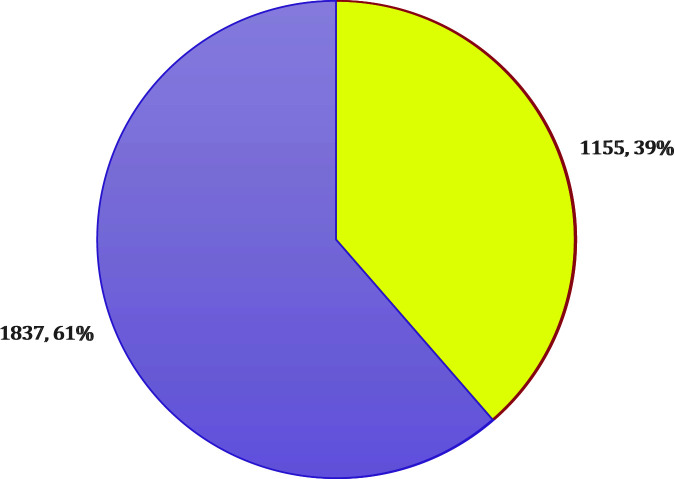
Nationality distribution of adult patients diagnosed with movement disorders at Hamad General Hospital, Doha, Qatar (January 2014–March 2025): Qataris versus non-Qataris.

**Figure 3.**
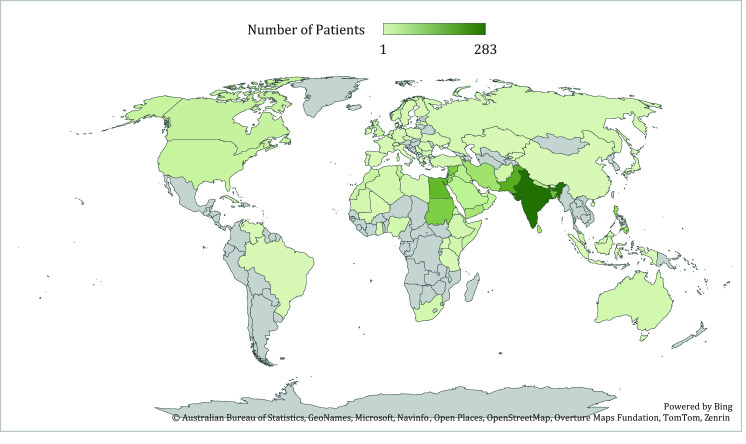
Geographic distribution of non-Qatari adult patients diagnosed with movement disorders at Hamad General Hospital, Doha, Qatar (January 2014–March 2025).

A total of 3017 adult patients were evaluated (Figure 1). Tremor was the most frequent diagnosis (1631 cases, 54%), followed by PD (778 cases, 25.8%), while dystonia and neuropathy each accounted for approximately 10% of cases. Using 2023 Planning and Statistics Authority data, the mid-year adult population denominator was estimated at approximately 2.5 million. The resulting crude prevalence estimates were 65 per 100,000 for tremors, 31 per 100,000 for PD, and approximately 12 per 100,000 each for dystonia and neuropathy, yielding an overall crude prevalence of approximately 121 per 100,000 adults. Qataris comprised 39% of the cohort (Figure 2), with the remaining 61% representing 78 nationalities, predominantly from South Asia (Figure 3).

### 3.2 Demographics of the tremor’s cohort

**Figure 4.**
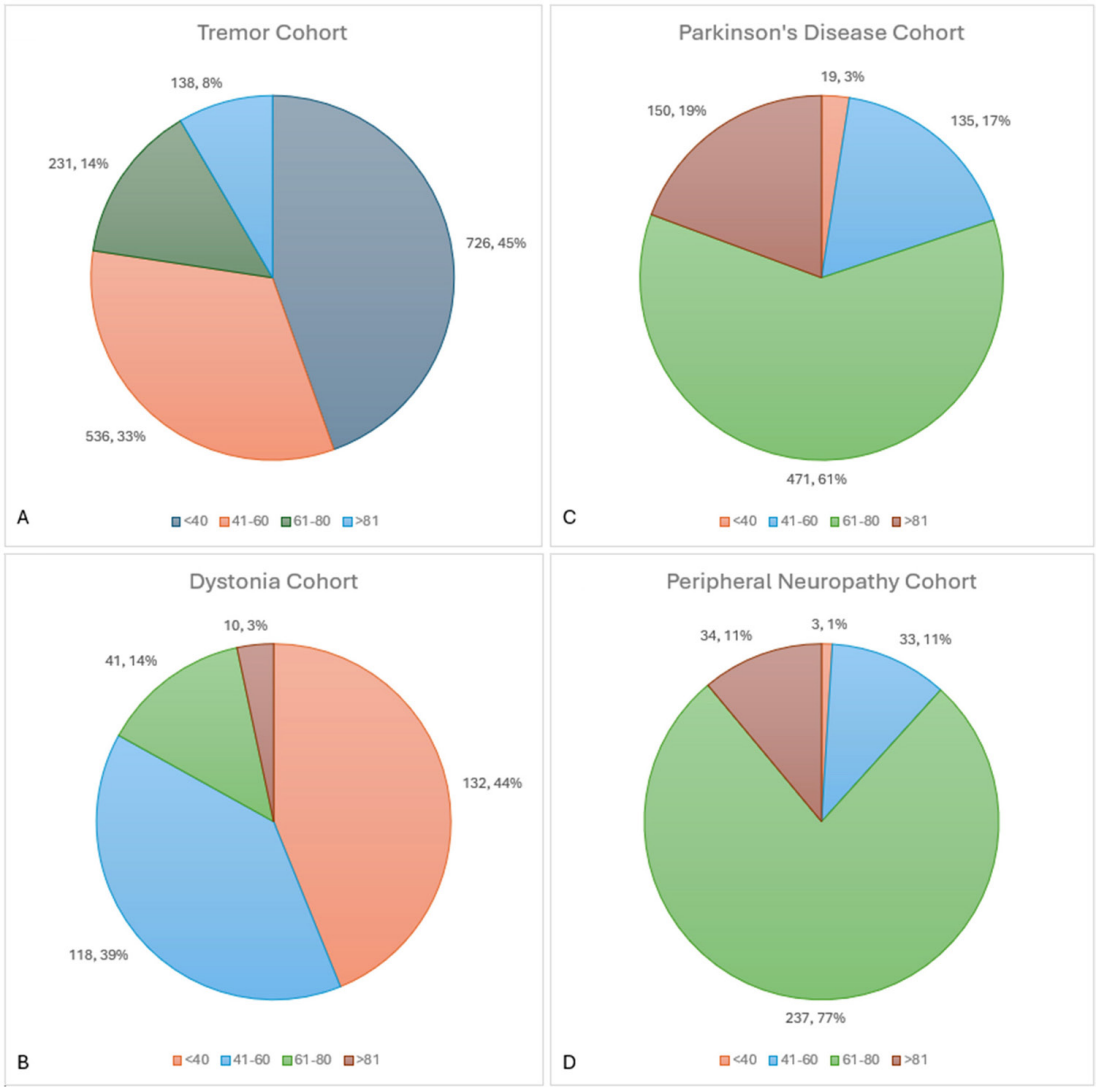
Age distribution (years) of adult patients across movement disorder cohorts at Hamad General Hospital, Doha, Qatar (January 2014–March 2025): (A) Tremor cohort, (B) Dystonia cohort, (C) Parkinson’s disease cohort, and (D) Peripheral neuropathy cohort.

**Figure 5.**
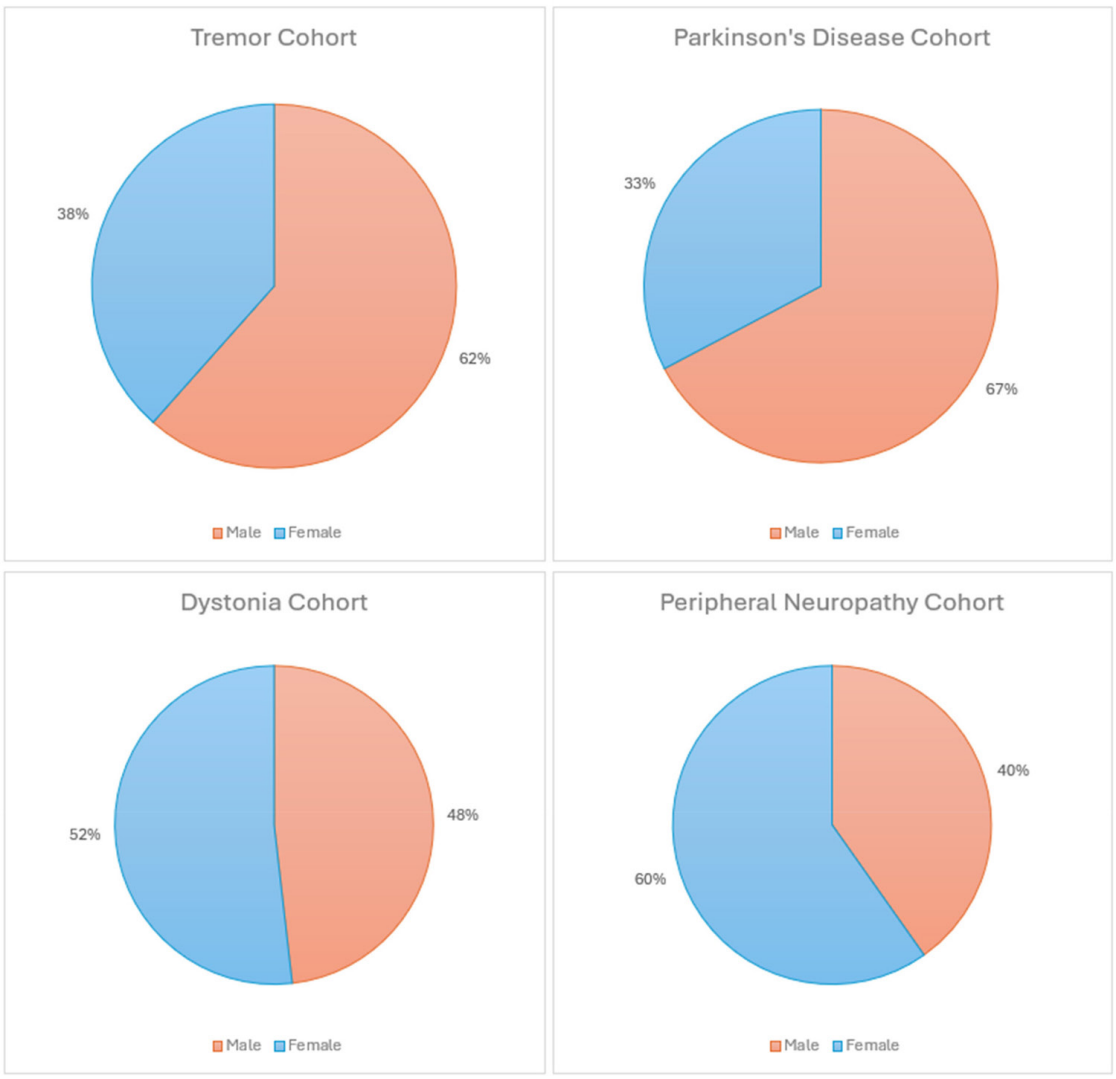
Sex distribution across movement disorder cohorts among adult patients at Hamad General Hospital, Doha, Qatar (January 2014–March 2025).

**Figure 6.**
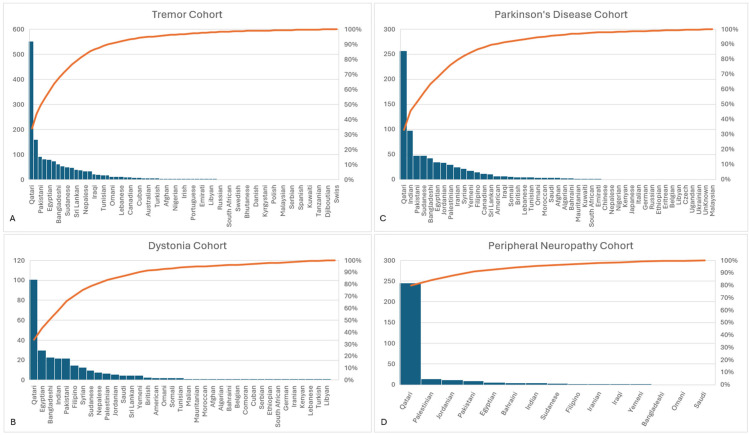
Nationality distribution across movement disorder cohorts among adult patients at Hamad General Hospital, Doha, Qatar (January 2014–March 2025): (A) Tremor cohort, (B) Dystonia cohort, (C) Parkinson’s disease cohort, and (D) Peripheral neuropathy cohort.

**Figure 7.**
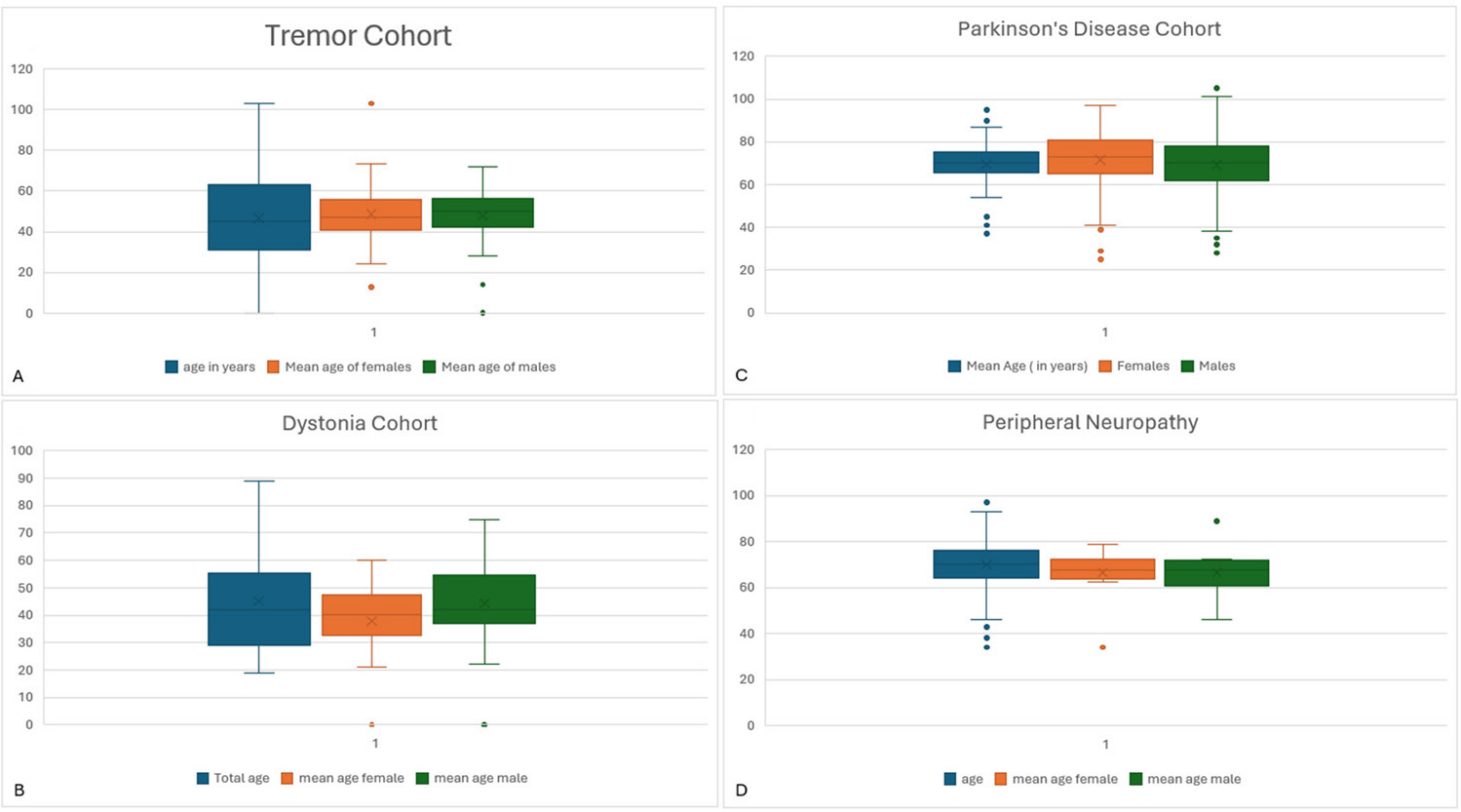
Age distribution by sex (scatter plots) across movement disorder cohorts among adult patients at Hamad General Hospital, Doha, Qatar (January 2014–March 2025): (A) Tremor cohort, (B) Dystonia cohort, (C) Parkinson’s disease cohort, and (D) Peripheral neuropathy cohort.

Qataris represented the highest proportion of patients diagnosed with tremors (33.7%). The Indian population ranked second at 9.7%, followed by the Pakistani population at 5.7% (Figure 4A). The distribution of patients diagnosed with tremors by gender showed a male predominance, with 62% of the cohort. A total of 78% of the patients were diagnosed with tremors before the age of 60 (Figures 5A and 6A). Only 22% were diagnosed after 60 years, of whom 8% were diagnosed with tremors beyond 81 years. The median age of patients with tremor was approximately 50 years (interquartile range [IQR], 30–70). Females had a median age of approximately 45 years, while males had a median age of approximately 47 years, with similar distribution patterns (Figure 7A).

### 3.3 Demographics of PD cohort

Qataris represented the highest proportion of patients diagnosed with Parkinsonism (33%). The Indian population ranked second at 12.5%, followed by the Pakistani, Sudanese, and Bangladeshi populations, each at 6.1% (Figure 4B). The male population predominated among patients diagnosed with PD at 67%. A total of 78% of patients with PD were diagnosed between 41 and 80 years of age. Only 3% were diagnosed before 40 years, and 19% were diagnosed above 81 years (Figures 5B and 6B). The median age of patients with PD was approximately 70 years (IQR, 60–80). Females demonstrated a slightly higher median age than males, although overall age distributions were comparable (Figure 7B).

### 3.4 Demographics of the dystonia cohort

Qataris represented the highest proportion of patients diagnosed with dystonia (33.5%). The Egyptian population ranked second at 10%, followed by the Pakistani, Indian, and Bangladeshi populations, each at 7% (Figure 4C). A total of 48% of the patients with dystonia were males. About 44% of patients were diagnosed below 40 years of age. Nearly half of the patients (53%) were diagnosed between 41 and 80 years of age, and only 3% were diagnosed above 81 years of age (Figures 5C and 6C). The median age of patients with dystonia was approximately 45 years (IQR, 30–60), with broadly similar age distributions between males and females (Figure 7C).

### 3.5 Demographics of the neuropathy cohort

Qataris represented the highest proportion of patients diagnosed with neuropathy (79.5%). The Palestinian population ranked second at 4.5%, followed by the Jordanian population at 3.5% (Figure 4D). A total of 40% of the patients diagnosed with peripheral neuropathy were males. While less than 1% were diagnosed below 40 years of age, 88% of the patients were diagnosed between 41 and 80 years. Only 11% were diagnosed at an age above 81 years (Figures 5D and 6D). Patients with neuropathy had a median age of approximately 70 years (IQR, 60–80), with similar age distributions across sexes (Figure 7D).

## 4. DISCUSSION

### 4.1 Overall

This study describes the distribution of movement disorders in a demographically diverse adult population in Qatar. Tremor was the most frequently recorded diagnosis, followed by PD, while dystonia and neuropathy were less common. This pattern aligns with international epidemiological data, in which tremor is typically the most prevalent movement disorder, and PD prevalence increases with age.^7,8^ The crude prevalence estimates observed in this cohort fall within the lower range of global reports, likely reflecting Qatar’s relatively young population structure. Similar demographic patterns have been reported in other GCC countries, where healthcare systems serve predominantly working-age expatriate populations. Estimates from the Global Burden of Disease 2019 study indicate that the age-standardized point prevalence of PD in the MENA region was 82.6 per 100,000 in 2019, with national estimates ranging from 63.6 to 119.3 per 100,000.^9^ Differences in case ascertainment and modelling approaches, however, limit direct comparison with single-center clinical cohorts.

### 4.2 Distribution according to nationality

The multinational composition of the cohort reflects Qatar’s demographic structure, where expatriate workers constitute a substantial proportion of the resident population.^7,11^ Overall, among the movement disorders, Qataris were the largest proportional representation in the incidence, especially in the neuropathy grouping, where they represented almost 80% of all cases. Of note, differences between Qataris and patients from other nationalities may reflect genetic predispositions (higher consanguinity among Qataris), occupational exposures (manual labor vs sedentary work), and healthcare access/referral pathways. These factors may explain variations in disease burden beyond pure epidemiology. Reflecting the country’s reliance on a predominantly transient expatriate workforce, most expatriate residents are required to leave the country upon reaching retirement age, resulting in a population with a disproportionately small elderly non-Qatari segment.

### 4.3 Distribution according to sex

A clear pattern emerged across the different disorders: men were affected more often, a finding that should be interpreted in light of Qatar’s uniquely skewed population structure, where males comprise the majority of the total population due to a predominantly male expatriate workforce.^11^ This was particularly the case for tremors and PD, in which 62% and 67% of the cohorts were male patients, respectively. This male predominance is not limited to Qatar; it has been consistently reported in studies across other nationalities, with Parkinson’s being about 40% more common in men.^7^ The average age of diagnosis for PD in our sample was just under 70 years, which is in line with international data showing the risk of developing PD increases sharply with age.^8^

### 4.4 Distribution according to age

Age distribution varied markedly across movement disorder categories. Most patients with tremors were diagnosed before the age of 60 years, suggesting earlier symptom recognition or referral patterns for tremor-related complaints. In contrast, PD was predominantly diagnosed between 41 and 80 years, with a median age close to 70 years, which is consistent with population-based studies and meta-analyses demonstrating a sharp increase in PD incidence and prevalence with advancing age, particularly after the sixth decade of life.^12^ Patients with dystonia exhibited a younger age profile, with a median age in the mid-40s, in keeping with epidemiological studies showing that many forms of primary dystonia present in early to mid-adulthood.^13,14^ Neuropathy cases in this cohort were concentrated in older age groups, with most diagnoses occurring between 60 and 80 years, aligning with global data indicating that peripheral neuropathy prevalence increases substantially with age and is strongly influenced by age-related comorbidities such as diabetes.^13^ Together, these findings highlight distinct age-related patterns across movement disorder subtypes that are consistent with previously published epidemiological evidence.

### 4.5 Limitations

This study has several limitations. First, its retrospective design relies on existing clinical documentation, which may be subject to incomplete data capture, variability in diagnostic coding, and inconsistencies in clinical classification across providers. Second, the analysis was limited to adult patients evaluated at a single tertiary referral center, which may introduce referral bias and limit generalizability to patients managed in private, military, or non-tertiary healthcare settings. Third, the unique demographic structure of Qatar, characterized by a predominantly transient expatriate workforce with a relatively small elderly non-Qatari population, may influence age- and nationality-specific distributions and limit direct comparison with populations that have more stable age structures. In addition, peripheral neuropathy was included because some patients were referred to the movement disorders clinic due to associated gait disturbance, balance impairment, or tremor-like symptoms; however, neuropathy is not a classical movement disorder, and its inclusion may represent an expanded interpretation of the movement disorder spectrum and contribute to diagnostic heterogeneity. Finally, the lack of consistent documentation of tremors and dystonia subtypes, exclusion of pediatric cases, and absence of systematic confirmatory investigations (such as genetic testing or standardized imaging review) reflect inherent constraints of retrospective data and may affect diagnostic granularity.

## 5. CONCLUSION

While the retrospective single-center design and reliance on routine clinical documentation impose important limitations, the findings provide region-specific epidemiological insight from a setting that remains underrepresented in the literature. These data may serve as a foundation for future prospective, population-based investigations incorporating standardized diagnostic criteria and expanded clinical characterization. AI was used for grammatical revision and was not used for synthesis of any of the literature or figurative material.

## ACKNOWLEDGMENTS

We thank the neurosurgery and neuroinstitute department for facilitating this work through infrastructure, guidance and commitment to research and continuous medical education.

## AUTHOR CONTRIBUTIONS

AM: Conceptualization, study design, data interpretation, manuscript drafting, critical revision, supervision, and final approval of the manuscript. AA: Data collection, data organization, literature review, manuscript drafting, and final approval of the manuscript. FA-S: Data collection, data verification, manuscript drafting, and final approval of the manuscript. ZAM: Literature review, methodological support, manuscript revision, and final approval of the manuscript.IA: Data analysis support, interpretation of epidemiological findings, manuscript revision, and final approval of the manuscript. AA: Manuscript revision, methodological input, administrative support, and final approval of the manuscript.

## DATA AVAILABILITY

Data is available with the authors upon request and is subject to approval for sharing from HMC.

## CONFLICT OF INTEREST

The authors declare that they have no known competing financial interests or personal relationships that could have appeared to influence the work reported in this article.
